# Incomplete Conflict of Interest Disclosures

**DOI:** 10.1001/jamanetworkopen.2019.16040

**Published:** 2019-10-25

In the Original Investigation titled “Rate of Fentanyl Positivity Among Urine Drug Test Results Positive for Cocaine or Methamphetamine,”^1^ published April 26, 2019, the Conflict of Interest Disclosures statement for Robert K. Twillman, PhD, was incomplete. The complete disclosure statement is as follows: “Conflict of Interest Disclosures: Drs LaRue, Dawson, Frasco, Huskey, and Guevara and Mr Whitley are employees of Millennium Health, San Diego, California. Dr Twillman reports being a consultant for Millennium Health; having been employed by the Academy of Integrative Pain Management (from September 2010 through January 2019), which received funding from multiple commercial interests related to opioids and pain management; and having previously participated as a paid consultant, speaker, and author for educational programs and a peer-reviewed publication related to drug monitoring programs and public policy related to pain management, which were sponsored by manufacturers of opioids or other medications for pain management.” This article has been corrected.^1^
